# Generalized direct fabrication of embedded–magnet microrobots with enhanced material compatibility

**DOI:** 10.1007/s12213-026-00208-8

**Published:** 2026-07-08

**Authors:** Yang Yang, Jeremy B. Gan, Jialong Huang, Sven Mucke, Aaron C. Davis, Haiyan Wang, David J. Cappelleri

**Affiliations:** 1 Multi-Scale Robotics and Automation Lab, School of Mechanical Engineering, Purdue University, West Lafayette, IN 47906 USA; 2 Weldon School of Biomedical Engineering, Purdue University, West Lafayette, IN 47906 USA; 3 School of Materials Engineering, Purdue University, West Lafayette, IN 47906 USA; 4 Elmore Family School of Electrical and Computer Engineering, Purdue University, West Lafayette, IN 47906 USA; 5 Purdue Institute for Cancer Research, Purdue University, West Lafayette, IN 47906 USA

**Keywords:** Microrobot, Fabrication, Surface modification, Sputtering, TPP

## Abstract

Magnetically actuated microrobots offer transformative potential for biomedical applications such as targeted drug delivery and minimally invasive diagnostics. However, existing fabrication methods are constrained by challenges in magnetic material integration, structural robustness, and reproducibility. In this work, we present an improved direct-printing strategy that integrates permanent micro-magnets into microrobots during the two-photon polymerization (TPP) process, thereby eliminating the need for post-assembly alignment or insertion. To enhance magnetic-material compatibility and interfacial reliability, a sputtering-based surface modification technique is introduced, enabling robust integration of both pre-coated and surface-treated magnets. Using this approach, four functional microrobotic platforms are demonstrated: (1) a helical microswimmer for efficient propulsion, (2) a micro-scale tumbling microrobot for terrain locomotion, (3) a compliant micro-gripper for precise grasping and manipulation, and (4) a mini-MicroTumbler (MMT) incorporating a sputter-modified magnet for stable microscale actuation. Performance characterization was conducted under varying actuation frequencies and environments. The microswimmer exhibited frequency-dependent propulsion consistent with magnetic step-out behavior, the MicroTumbler achieved stable locomotion across inclined surfaces, the micro-gripper demonstrated controllable deformation and object manipulation, and the MMT showed reliable frequency-dependent motion. This study establishes a scalable, material-flexible, and high-fidelity fabrication method for embedded-magnet microrobots, broadening the design space and enabling the next generation of multifunctional, magnetically actuated microsystems.

## Introduction

Microrobots have emerged as powerful tools for various biomedical, environmental, and engineering applications due to their miniature sizes, controllable motion, and ability to perform precise tasks in constrained environments. These robots, typically ranging from a few micrometers to millimeters in size, have significant potential in targeted drug delivery [1, 2], minimally invasive surgery [3, 4], environmental remediation [5], and microscale assembly operations [6, 7].

Currently, the fabrication of microrobots often involves a multi-step process. Recently, we proposed Helical Adaptive Multi-material MicroRobots (HAMMRs) with fast translational velocity for adaptive locomotion [8, 9] and the Helical Multi-Material MicroRobot with a Detachable Payload (HMMR-DP) [10]. Structures are first printed or constructed using methods such as soft lithography, 3D printing, or photolithography. Subsequently, these structures are magnetized by either embedding magnetic particles post-fabrication or through a separate magnetization process. While these methods have enabled significant advancements, they often suffer from issues such as misalignment of magnetic materials, inconsistent magnetic properties, and complicated manufacturing workflows.

Among various microfabrication technologies, Two-Photon Polymerization (TPP) stands out due to its unique advantages, including sub-micron resolution [11], high design flexibility [12], and the capability to fabricate complex 3D structures with precision [13, 14]. TPP enables precise control over structural details, which is critical for microrobotic systems that demand intricate geometries and reliable functional properties.

However, despite the advantages offered by TPP, existing fabrication and assembly methods for magnetically functionalized microrobots remain cumbersome and inefficient. Common challenges include the necessity for multiple fabrication steps [15, 16], difficulties in embedding or aligning magnetic components [17, 18], and reduced scalability and reproducibility due to manual assembly processes [19]. These limitations significantly hamper the broader adoption and practical utility of microrobots.

An additional challenge arises from the surface incompatibility between magnetic materials and photoresists, which can lead to poor adhesion or structural defects during direct printing. Commercial micro-magnets are often supplied with protective coatings, such as nickel [20, 21] or gold [22], that provide sufficient bonding strength with the photoresist. However, uncoated magnets typically exhibit low surface energy and chemical inertness [23, 24], making them less suitable for direct integration. To address this issue, surface modification techniques—such as plasma treatment [25], or thin-film deposition [26]—can be employed to improve interfacial adhesion. Among these methods, sputtering offers a clean, controllable, and low-temperature process capable of depositing a thin, uniform layer that enhances chemical compatibility between the magnet and the polymer resin with negligible impact on the magnet’s properties or geometry [27–29]. This modification ensures stable bonding during the TPP process and broadens the range of magnetic materials that can be directly integrated into microrobots.

In this paper, we propose a novel fabrication method that overcomes the current limitations by directly integrating magnetic materials into microrobots during the TPP printing process itself. Unlike traditional multi-step fabrication and magnetization methods, our approach combines magnet integration seamlessly with microrobot construction in a single fabrication step. This innovation not only simplifies the fabrication workflow but also ensures precise alignment, uniform magnetic properties, and scalability, ultimately facilitating enhanced functionality and expanding the practical applications of microrobots.

**Fig. 1:**
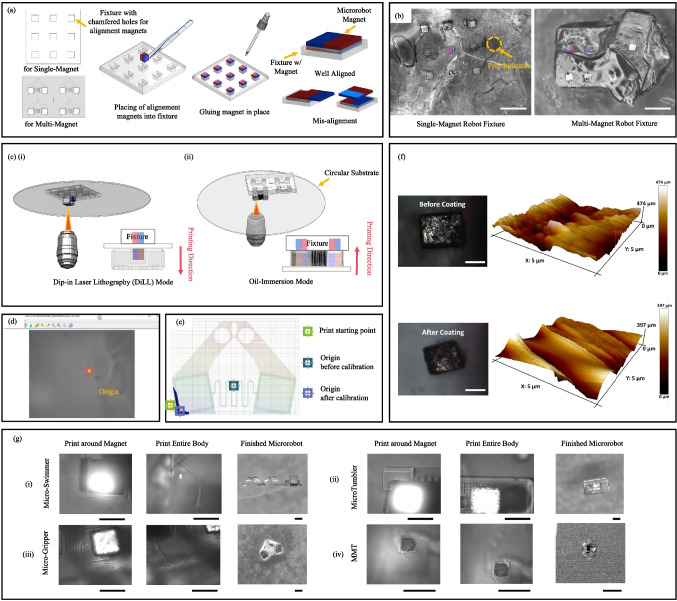
Fabrication process and printing strategies of magnetically embedded microrobots. (**a**) Schematic of customized fixture preparation. Predefined chamfered molds are printed using IP-Q, into which magnets are manually inserted with tweezers and fixed using resin. Proper alignment between the fixture magnets and microrobot magnet is critical to ensure consistent magnet orientation during printing. (**b**) Optical images of the actual printed fixtures for single-magnet and multi-magnet microrobot fabrication. The magnetic pole of the inserted magnet is labeled for reference. The pole indicator denotes the magnet orientation. Scale bars: 1 mm. (**c**) Comparison of the two-photon polymerization printing configurations: (i) Dip-in Laser Lithography (DiLL) mode and (ii) Oil-Immersion mode, highlighting the printing direction and the spatial relationship between the fixture, substrate, and objective lens. The tumbler and micro-gripper microrobots are used as printing examples for DiLL and Oil-Immersion mode, respectively. (**d**) Cross-shaped reference marker used for origin calibration in the camera software interface (Manta G-507B, Allied Vision). (**e**) Offset calibration strategy used to align the design’s print starting point with the true origin. (**f**) Optical and atomic force microscopy (AFM) images of uncoated magnets before and after sputtering. The optical micrographs show the surface condition, while the AFM surface profiles confirm reduced roughness and improved uniformity after surface modification. Scale bars: 100 $\mu$m. (**g**) Sequential fabrication images of three microrobot types ((i) micro-swimmer, (ii) MicroTumbler, (iii) micro-gripper, and (iv) mini-MicroTumbler (MMT), showing the process of printing around the magnet, completing the body, and the final released microrobot. Scale bars: 250 $\mu$m

## Microrobot fabrication

In this section, we describe the fabrication process developed for magnetically actuated microrobots, employing an innovative direct-printing approach integrated with customized fixtures. Two primary fabrication scenarios are then presented: microrobots with a single embedded magnet, and microrobots containing multiple magnets arranged with controlled magnetic polarities.

### Fixture selection

Customized fixtures were fabricated from IP-Q photoresist using a Photonic Professional GT2 two-photon 3D printer (Nanoscribe GMBH) with a 10$\times$ objective lens, chosen to accommodate fixture dimensions adequately. These fixtures featured predefined molds with a chamfer into which magnets were manually positioned using precision tweezers. To securely anchor the magnets within the fixture, a small drop of IP-Q resin was applied onto the inserted magnets and subsequently cured using UV light. During this gluing step, the fixture was placed on a flat surface to ensure that only the upper surface of the magnet came into contact with the resin, while the bottom surface remained flush with the substrate. This prevents the formation of a spacer layer beneath the magnet and ensures tight contact with the substrate—a critical factor for accurate alignment and stable printing. Figure 1(a) illustrates the fixture design used in this study. By leveraging the fixture-based approach for magnet placement, arbitrary magnet configurations, including multiple magnets with varying orientations and spatial arrangements, can be systematically integrated into microrobot structures. The fixtures were configured to accommodate nine individual magnets or four grouped magnet pairs, enabling the potential of batch fabrication of microrobots within a single print job. The minimum center-to-center spacing between magnets in fixture was set to approximately 450 $\mu$m to provide sufficient clearance for polymer structure printing, thereby preventing overlap or interference between adjacent robots. An example fixture developed for single-magnet microrobot fabrication is illustrated. Proper magnet orientation is critical for microrobot actuation. To ensure clear identification of pole direction, we used a marker to denote the south-to-north direction on the fixture (Fig. 1(b)). This served as a reference for orienting the circular substrate during printing to maintain correct spatial alignment between the magnet and the robot’s geometry. After loading, the magnets were inspected under a microscope to verify alignment accuracy. Only well-aligned magnets—those with the desired magnetic axis orientation—were retained, as misalignment can lead to unintended actuation behavior. Figure 1(b) shows the actual fabricated fixture used in this study.

### Two-photon polymerization (TPP) printing modes

The Nanoscribe system offers two distinct modes of two-photon polymerization: Dip-in Laser Lithography (DiLL) and Oil-Immersion mode.

In DiLL mode, the objective lens is directly immersed into the liquid photoresist, which simultaneously functions as the optical immersion medium. In this approach, the printing substrate is positioned between the fixture and the magnet onto which the microrobot is fabricated (Fig. 1(c)(i)). The primary advantage of DiLL mode is its ability to print microrobots with heights significantly exceeding the magnet’s dimensions, due to the larger available vertical printing range. However, magnet alignment in DiLL mode can be more challenging, especially for the multi-magnet microrobots, as the magnetic attraction force is notably reduced by the separation introduced by the substrate, making precise alignment more difficult.

In contrast, oil-immersion mode involves placing a droplet of photoresist onto a circular coverslip, beneath which immersion oil is applied to create an optical coupling between the substrate and objective lens. In this configuration, both the fixture and the magnet are located on the same side of the substrate and are in direct contact with each other, providing superior magnet visibility and facilitating precise alignment (Fig. 1(c)(ii)). This is essential to ensure that magnets are securely positioned and isolated from each other, minimizing magnetic interference and maintaining consistent actuation performance. However, since microrobots are fabricated in the confined space between the substrate and the fixture, the vertical height of the printed structures should not substantially exceed the magnet height by more than approximately 50 $\mu$m, as taller structures risk colliding with the fixture during the printing process. Despite this spatial constraint, clean detachment of the printed microrobot is reliably achieved after development, primarily due to surface tension between the robot base and the substrate, which holds the microrobot in place while the fixture is removed.

In our study, both printing modes were strategically employed based on specific microrobot design requirements: DiLL mode was selected for fabricating single-magnet microrobots, where structural dimensions significantly exceeded the magnet height. In contrast, Oil-Immersion mode was preferred for fabricating multi-magnet microrobots, where precise alignment, accurate magnet positioning, and controlled polar orientations were critical.

### Printing parameter optimization

To securely embed the magnets within the microrobot structure, a shell-like geometry was specifically designed. Despite manufacturer assurances of uniform magnet dimensions, slight deviations commonly occur at the microscale, significantly affecting fabrication accuracy. To accommodate such variations, the shell dimensions were intentionally designed to be slightly larger than the nominal magnet size. This design ensures reliable magnet embedding, despite dimensional inconsistencies. Following the development step, residual uncured resin may remain within the shell cavity. To address this, post-processing UV exposure was performed to cure any residual resin, thereby tightly securing the magnet inside the polymeric structure.

In the Nanoscribe system, the origin is defined as the spatial position where the user initiates the printing process by pressing the “Start” button. However, the actual starting point of the printed structure can differ significantly from this designated origin due to the complexity of the robot geometry and internal design features. Therefore, accurate identification and calibration of the printing origin were essential to precisely encapsulate the magnets within the polymeric robot structures.

To address the absence of explicit origin markers within the Nanoscribe graphical interface, we implemented a calibration procedure. Specifically, we first printed a cross-shaped reference marker onto the substrate, ensuring its geometric center aligned exactly with the defined origin (Fig. 1(d)). By visually identifying and digitally marking this cross on-screen, a consistent, accurate, and reproducible printing reference point was established for subsequent fabrication runs.

Due to intricate robot designs, the origin position and actual print-start location frequently differ, necessitating careful tracking of the offset between these two points. Precisely determining this spatial relationship was critical to guarantee correct magnet encapsulation within the intended microrobot geometry. To simplify this alignment challenge, one corner of the embedded magnet (or equivalently, the corresponding corner of the designed shell structure) was consistently designated as the origin during design and printing. Using straightforward geometric relationships, the necessary stage movements to ensure accurate positioning were calculated according to the following equations:

1 $$\begin{aligned} \begin{aligned} X_{stage} = X_{origin} + X_{offset} \\ Y_{stage} = Y_{origin} + Y_{offset} \end{aligned} \end{aligned}$$

where $X_{stage}$ and $Y_{stage}$ represents the adjusted coordinates for the printing stage to ensure the defined origin precisely aligns with the magnet’s designated corner (Fig. 1(e)). Through this calibration and offset procedure, we consistently achieved accurate and reliable magnet placement within the microrobot structures.

In this work, IP-S photoresist was selected for robot fabrication due to its compatibility with the 25x objective lens, low shrinkage, high mechanical stability, excellent surface finish, and proven biocompatibility [30]. Careful optimization of printing parameters was crucial to avoid bubble formation during the direct laser exposure of the magnet surface. Through empirical testing, optimal printing parameters were determined. It was established that the default DiLL and oil-immersion parameters provided by the Nanoscribe system—specifically, laser power of 50 mW and scanning speed of 100k $\mu$m/s—were sufficient to produce bubble-free, high-quality microrobots, given the protective coating of the micro-magnets.

Following printing, the fabricated samples were developed to remove unpolymerized resin. The development process consisted of immersing the samples in Propylene glycol methyl ether acetate (PGMEA) for 20 minutes, followed by rinsing with Isopropanol (IPA) for 30 seconds. After development, additional UV exposure was applied when necessary to ensure thorough curing. Finally, microrobots were carefully detached from the substrate using precision tweezers.

### Case one: microrobots with a single pre-coated magnet

Microrobots powered by magnetic fields typically rely on a single embedded magnet or a uniformly magnetized body to achieve locomotion, manipulation, and steering within micro-scale environments. To demonstrate the capability of our direct magnet integration approach, we first fabricated a simple microrobot embedding a single magnet.

Micro-magnets utilized in this case were obtained directly from SuperMagnetMan. To mitigate potential bubble formation during laser exposure, nickel-coated NdFeB cubic permanent magnets with standardized dimensions of 250 $\mu$m were selected.

Figure 1(c)(i) illustrates the fabrication setup for single-magnet microrobots (exemplified here by a magnetic tumbling microrobot) using the Dip-in Laser Lithography (DiLL) mode. In this configuration, a customized fixture containing multiple predefined molds is placed above a transparent substrate. The objective lens, immersed directly into the liquid photoresist below the substrate, polymerizes the structure around the magnet, encapsulating it precisely within the microrobot.

Using this fabrication strategy, two distinct robot types— swimming microrobots (Fig. 1(g)(i)) and tumbling microrobots (Fig. 1(g)(ii)— were successfully printed and will be presented in subsequent sections.

### Case two: microrobots with multiple pre-coated magnets

Building upon the successful demonstration of single-magnet microrobots, our approach was extended to fabricate microrobots integrating multiple magnets with identical polar orientations. Such multi-magnet arrangements significantly enhance the microrobot’s manipulation capabilities, directional control, and complexity of achievable motion, beneficial for precision manipulation. Micro-magnets utilized in this case were also the nickel-coated NdFeB cubic permanent magnets with standardized dimensions of 250 $\mu$m.

Figure 1(c)(ii) presents a schematic of the fabrication process for multi-magnet microrobots using the oil-immersion mode. In this configuration, both the fixture and magnets are positioned on the same side of the substrate, with the printing space located between the substrate and the fixture. This arrangement facilitates more accurate magnet alignment and ensures proper encapsulation within the polymer body.

Using this method, we successfully fabricated a functional magnetic micro-gripper (Fig. 1(g)(iii)), demonstrating the practical versatility and scalability of our direct-printing strategy for creating more complex, multi-magnet microrobotic systems.

### Case three: microrobots with a single uncoated magnet

To further extend the applicability of the direct printing approach, we investigated the fabrication of microrobots incorporating uncoated permanent magnets. In this case, commercially supplied 100 $\mu$m cubic NdFeB micro-magnets without surface protection were first coated in-house using a sputtering process to enhance their surface stability during two-photon polymerization.

The commercially supplied micro-magnets were coated with a 0.5 $\mu$m tantalum (Ta) layer using DC magnetron sputtering (Ta, 99.99$\%$) in an AJA International ATC-2000 system. Tantalum was selected for its biocompatibility, corrosion resistance, and paramagnetic properties, which make it suitable for biomedical microrobotic applications. Prior to deposition, the sputtering chamber was evacuated to a base pressure of $1 \times 10^{-7}$ Torr. Ta films were deposited at 300 W DC power and room temperature, with a pure argon (Ar) gas flow rate of 30 sccm, resulting in a working pressure of 5 mTorr during deposition.

Atomic Force Microscopy (AFM, Bruker Dimension Icon) surface topography maps (5 $\times$ 5 $\mu$m) and statistical analyses confirmed that the Ta coating effectively reduced nanoscale roughness (Fig. 1(f)). The average roughness (Ra) decreased from approximately 112 nm to 81.8 nm ($\approx$ 27$\%$ reduction), and the root-mean-square roughness (Rq) decreased from 137 nm to 112 nm ($\approx$18$\%$ reduction). More importantly, the coating significantly improved the print quality during two-photon polymerization (TPP). Prior to coating, bubble nucleation and edge retraction frequently occurred near the magnet surface, resulting in irregular or incomplete structures. After Ta deposition, the printed features were smooth, void-free, and dimensionally consistent.

We attribute this improvement to multiple interrelated effects of the sputtered Ta layer. The DC-sputtered Ta film rapidly forms a thin, high-energy oxide that enhances photoresist wetting; the dense metallic layer acts as an outgassing barrier, suppressing bubble formation during laser exposure and curing; and the metallic interlayer improves interfacial adhesion and stress tolerance under thermal and UV cycling. Collectively, the Ta coating not only smooths the surface morphology but, more critically, modifies the surface chemistry and transport properties, eliminating print-blocking failure modes observed on bare magnets and enabling reliable, reproducible fabrication of magnetically embedded microrobots.

After the surface modification, the fabrication procedure followed the same protocol established for the microrobot with pre-coated magnets. The fixture was mounted on one side of the substrate, and the microrobot body was printed directly around the magnet using the Dip-in Laser Lithography (DiLL) mode. Laser power and scanning speed were kept identical to those optimized in the pre-coated case. Using this method, we successfully fabricated a functional magnetic MMT incorporating a sputter-modified uncoated magnet, demonstrating reliable structural integrity, strong polymer–magnet bonding, and precise magnetic alignment (Fig. 1(g)(iv)).

## Results and discussion

### Fabrication performance analysis and comparison

Table 1 provides a comparison of the proposed fabrication strategy with representative methods for magnetically actuated microrobot fabrication, including post-assembly magnet insertion, magnetic particle embedding, and external magnetization approaches. Compared to post-assembly magnet insertion methods, which typically involve multiple fabrication and alignment steps, the proposed direct-printing approach integrates magnets during the fabrication process, significantly reducing assembly complexity and improving alignment accuracy. In contrast to particle embedding techniques, where magnetic particles are randomly distributed within a polymer matrix, our method enables the use of discrete permanent magnets with predefined orientation, resulting in stronger and more controllable magnetic actuation. Furthermore, particle-based methods often require high loading concentrations that negatively impact print resolution and mechanical properties, whereas the proposed approach preserves the intrinsic material properties of the photoresist.

Compared to external magnetization methods, which rely on bulk magnetization of magnetically responsive materials, the proposed strategy provides localized and programmable magnetic profiles through precise placement of embedded magnets. External magnetization is typically limited to composite materials containing magnetic particles and does not allow independent control of multiple magnetic domains within a single structure. In contrast, the fixture-based approach enables multi-magnet configurations with defined spatial arrangement and polarity, as demonstrated in the micro-gripper design.

In terms of fabrication time, the introduction of fixture-based magnet arrays enables automated, unattended batch fabrication, improving reproducibility and reducing manual intervention. Overall, the proposed method offers a unique combination of precise magnet integration, low fabrication complexity, and expanded material compatibility, distinguishing it from existing fabrication strategies while highlighting opportunities for future improvements in throughput and scalability.

**Table 1:** Comparison of Assembly Methods for TPP-Based Magnetically Actuated Microrobots

Method	Magnet		Fabrication	Alignment	Reference
	Integration		Complexity	Accuracy	
Post-assembly magnet insertion	Manual	$\geqslant$ 250 $\mu$m	High	Moderate	[10, 31]
Magnetic particle embedding	Coating, Distributed particles	$\leqslant$ 100 $\mu$m	Moderate	Low	[32–34]
External magnetization	Bulk magnetization	$\leqslant$ 100 $\mu$m	High	Low	[8, 35, 36]
Direct magnet printing	Pre-aligned embedded magnets	$\geqslant$ 100 $\mu$m	Low	High	This work

### Controlled navigation of the swimming microrobots

**Fig. 2:**
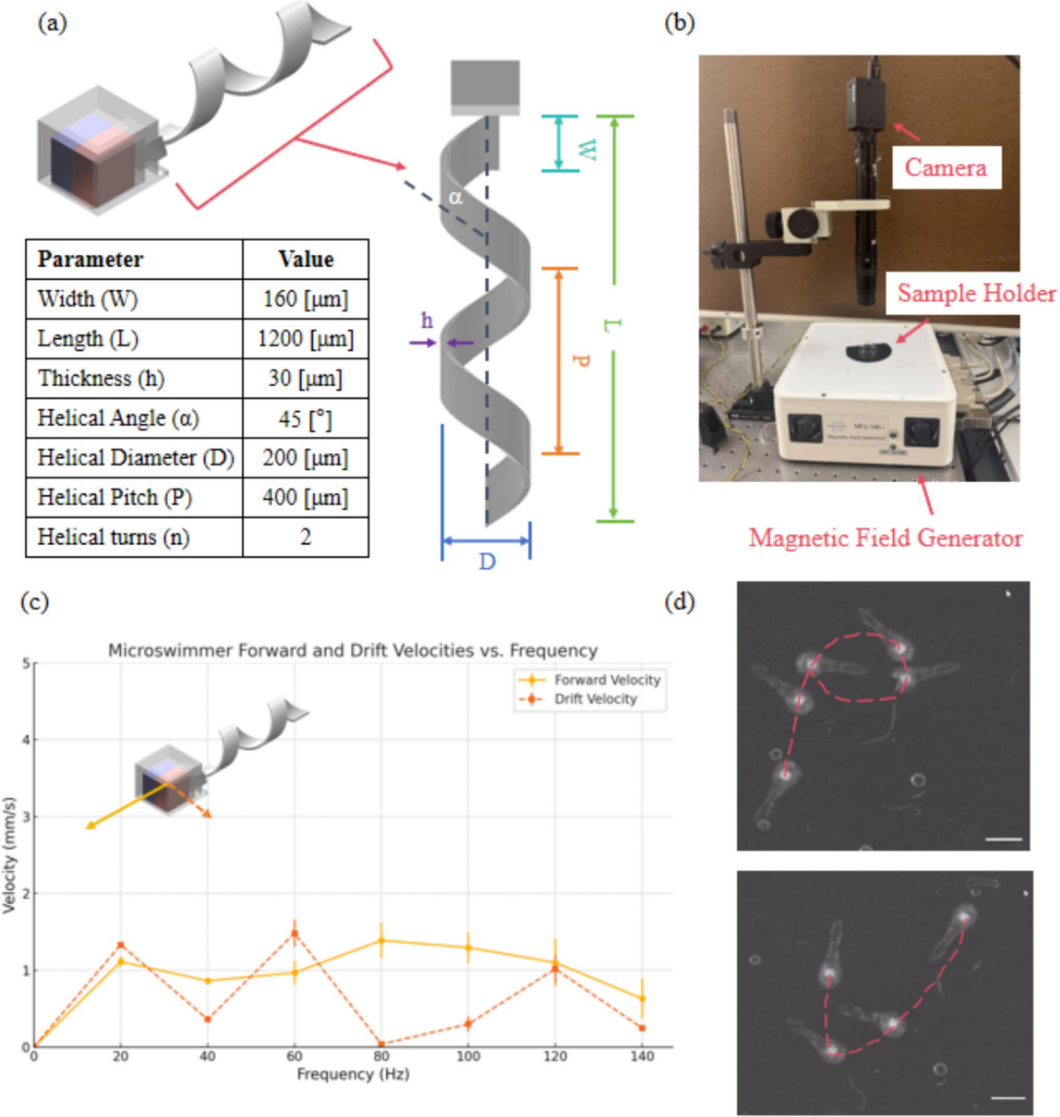
(**a**) Design schematic and structural parameters of the swimming microrobot. The microrobot consists of a magnetic head with a rigid helical tail. Key geometric parameters are listed in the accompanying table. (**b**) Experimental setup for frequency response testing. (**c**) Frequency response of forward and drift velocities. Maximum forward velocity is achieved at 80 Hz with minimal drift, suggesting optimal magnetic coupling (n = 3; error bars correspond to standard deviation (SD) across n samples). (**d**) Demonstration of programmable microrobot navigation. The swimmer was manually guided to trace the shapes of the letters “P” and “U” under 100 Hz actuation. Images are extracted from video recordings. Scale bars: 1 mm

Figure 2(a) provides detailed information regarding the geometric design of the swimming microrobot. The magnetic moment of the embedded magnet is aligned perpendicular to the helical axis, a common configuration that facilitates efficient propulsion under a rotating magnetic field.

In conventional microrobot fabrication, the fragile connection between the magnetic head and the flexible helical tail often poses challenges during post-printing magnet insertion. Such delicate assembly processes frequently result in structural damage or misalignment, lowering yield and reproducibility. In contrast, the direct-printing strategy introduced in this study significantly improves the structural integrity and assembly efficiency. By embedding the magnet during the printing process itself, the robot can be fabricated as a single cohesive structure with a much higher success rate.

Given that the 250 $\mu$m magnet embedded in the swimmer head is significantly stronger than those used in our previous designs, a higher-viscosity fluid was selected to provide increased damping and ensure more stable and controllable motion during testing. Therefore, as shown in Fig. 2(b), a sample holder filled with silicone oil (40 cSt, Alfa Aesar) was used to suspend the microrobot at the center of a magnetic field generator (MFG-100i, MagnebotiX), which provided a uniform magnetic field strength of 5 mT. The actuation frequency was manually adjusted to evaluate the microswimmer’s performance across a range of frequencies.

**Fig. 3:**
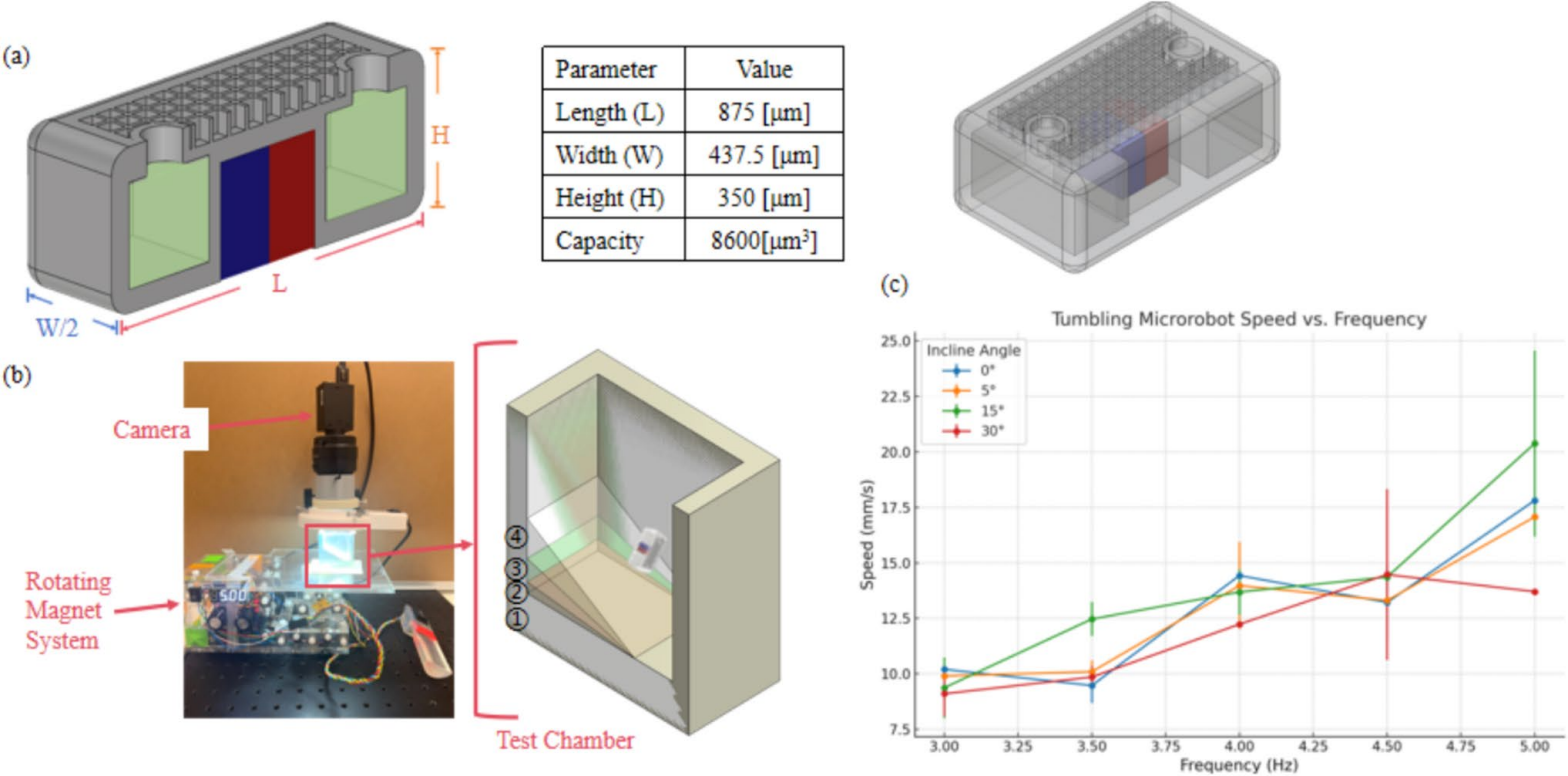
(**a**) Schematic and dimensions of the hollow MicroTumbler (section view). The robot features an internal cavity for drug loading (highlighted in green), with key design parameters summarized in the accompanying table. (**b**) Experimental setup for evaluating microrobot locomotion. The robot is placed inside a custom-built test chamber with inclined walls and actuated by an external rotating magnetic field. A camera is mounted above the chamber to record motion for speed analysis. The numbered surfaces correspond to different incline angles. (**c**) Quantitative results of tumbling speed as a function of actuation frequency across four incline angles (n = 3; error bars correspond to SD across n samples)

**Fig. 4:**
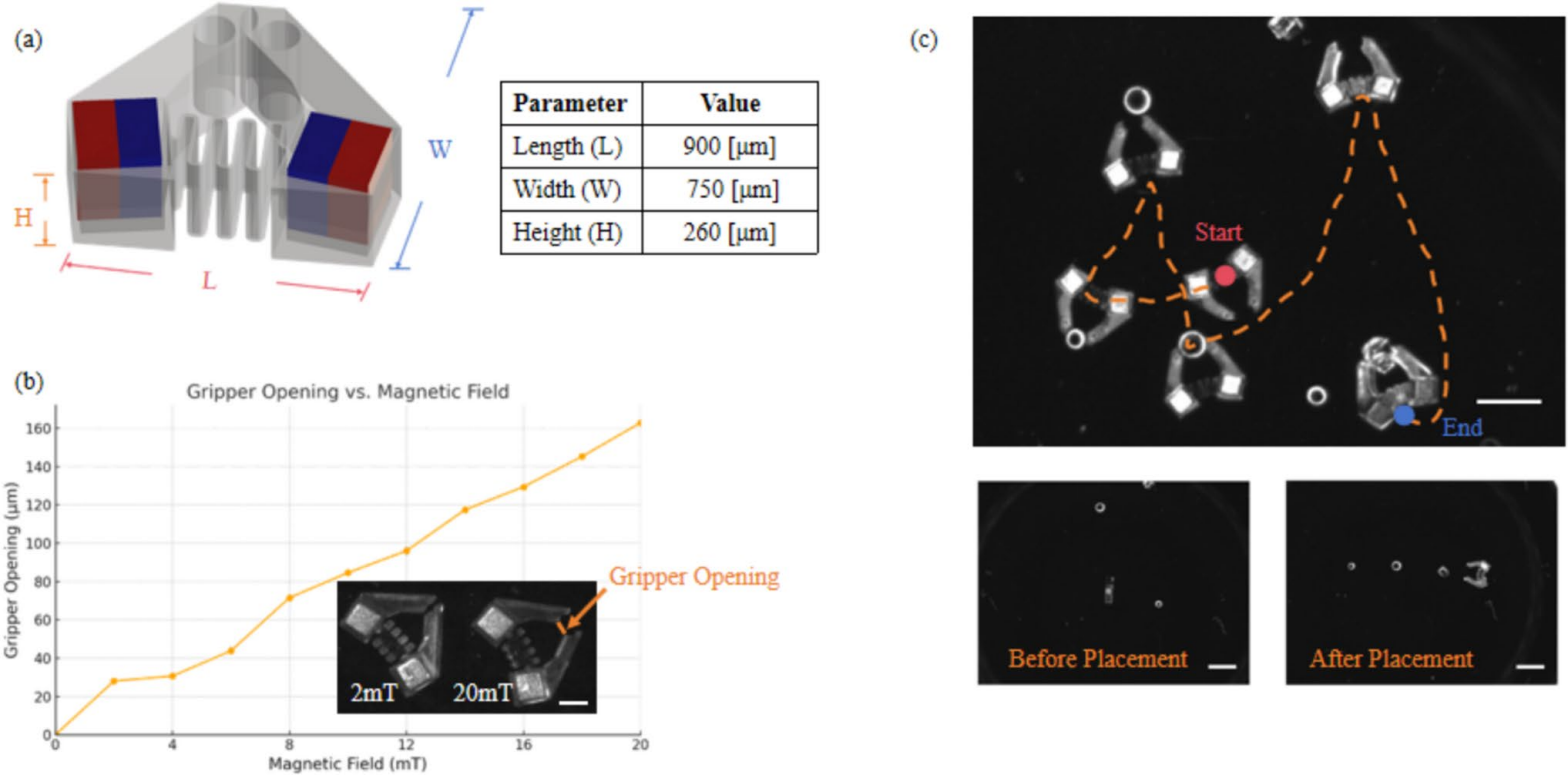
(**a**) Schematic of the magnetic micro-gripper design. Two oppositely magnetized blocks (red and blue) are embedded at an angle on either side of the gripper arms, which are connected by a compliant joint structure. Key geometric parameters are labeled. (**b**) Gripper opening as a function of magnetic field strength (n = 3). Scale bar: 250 $\mu$m. (**c**) Demonstration of object manipulation using the gripper. The micro-gripper successfully grasps and arranges objects of different shapes and sizes in a line. The manipulation path is highlighted by a dashed arrow. The red and blue points represent starting and ending points, respectively. Images are extracted from video recordings. Scale bar: 1 mm

**Fig. 5:**
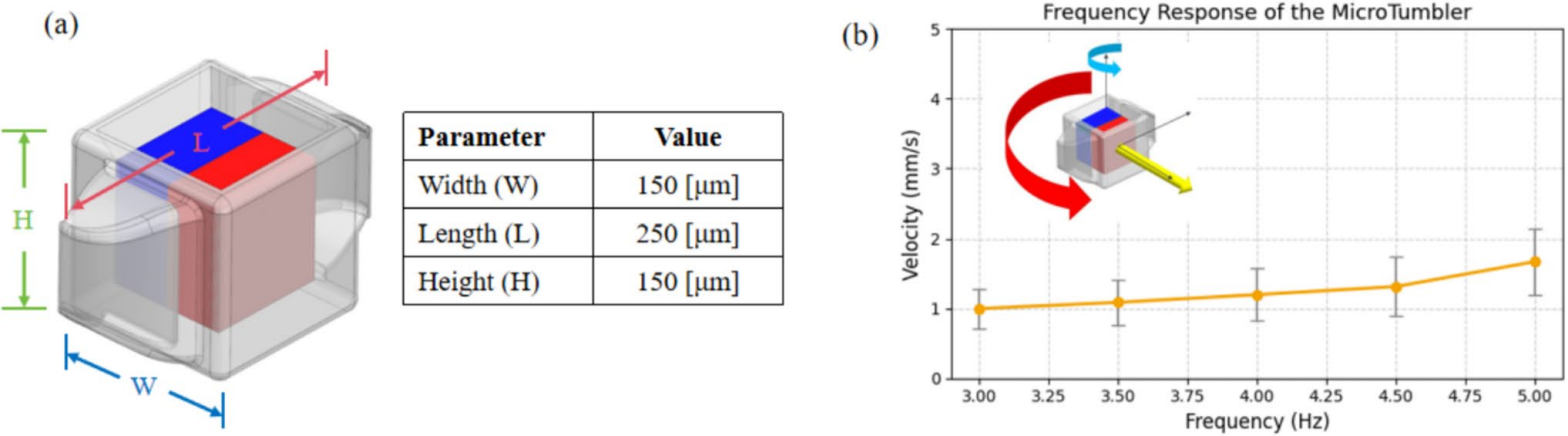
(**a**) Schematic and dimensional parameters of the MMT with an embedded sputter-treated magnet. (**b**) Frequency response of the MMT showing mean translational velocity and standard deviation as a function of actuation frequency in dry area (n = 3; error bars correspond to SD across n samples). The inset illustrates the direction of the rotating magnetic field (red), magnetic moment (yellow), and tumbling motion (blue)

All experimental results reported in Figs. 2, 3, 4 and 5 are based on three independent trials (n = 3). For each data point, three separate microrobots were tested, and each robot was used only once to avoid potential effects of fatigue, hysteresis, or surface wear during repeated actuation. Microswimmer performance was quantified using two metrics: forward velocity, defined as motion parallel to the direction of the rotating field, and drift velocity, representing deviation from the intended trajectory. The frequency-dependent responses are illustrated in Fig. 2(c). All experiments are conducted at room temperature of 20 $^{\circ }$C. At low frequency, drift velocity exceeded forward velocity, indicating significant wobble and inefficient propulsion at low torque. As the frequency increased, forward velocity peaked at 80 Hz, while drift decreased sharply, reflecting optimal torque transfer and stable forward motion. Beyond 80 Hz, a decline in forward velocity and rise in drift velocity indicated step-out behavior, where the microrobot fails to synchronize with the rotating field, leading to instability.

Although 80 Hz corresponded to the swimmer’s peak forward velocity, a slightly higher frequency of 100 Hz was selected for the navigation demonstration to achieve more stable control and enhanced visual tracking, enabling the swimmer to be effectively navigated along complex trajectories. To demonstrate controlled navigation, the swimmer was actuated at 100 Hz and 5 mT and manually guided to trace predefined shapes. As shown in Fig. 2(d), the swimmer successfully traced the letters “P” and “U,” demonstrating its potential for programmable path-following and targeted delivery applications.

### Frequency response of the MicroTumblers

Figure 3(a) illustrates the schematic design and operating principle of the MicroTumbler, adapted from our previous design framework [31, 37]. The MicroTumbler integrates a pre-coated permanent magnet for magnetic actuation and features a hollow cavity that can be used for drug loading. A grid structure is included for wax sealing, using temperature-sensitive wax to prevent premature release of the encapsulated therapeutic agents. The design enables controlled drug delivery in targeted environments. In this study, we investigated its locomotion behavior across varying actuation frequencies and incline angles to evaluate performance under different terrain conditions.

All experiments were conducted in a dry environment to eliminate fluidic damping and isolate mechanical interaction with the substrate. The MicroTumbler was placed inside a custom-built test chamber featuring an angled surfaces with inclinations of 0$^\circ$, 5$^\circ$, 15$^\circ$, and 30$^\circ$. It was actuated by a rotating magnetic field generated beneath the chamber. The actuation field was produced using a cylindrical NdFeB permanent magnet (2.54 cm diameter $\times$ 2.22 cm height, Cyl1875, SuperMagnetMan) mounted on a two-degree-of-freedom motorized holder (Fig. 3(b)). The magnet was rotated at discrete frequencies of 3, 3.5, 4, 4.5, and 5 Hz, inducing a magnetic torque that propelled the magnetized microrobot. The magnetic flux density at the microrobot’s location was estimated to range from 12.5 to 19.4 mT, depending on the orientation of the magnet relative to the robot’s magnetic moment [1]. A FLIR imaging camera equipped with a TAMRON lens was mounted directly above the chamber to capture real-time motion of the microrobot, while an AmScope fluorescent ring light (Model No. FRL8) was used to enhance visual contrast during recording. Figure 3(b) shows the physical and schematic setup used for the tests.

As shown in Fig. 3(c), the MicroTumbler’s speed generally increased with higher actuation frequencies across all incline angles. Standard deviations were generally low (typically less than 1 mm/s), indicating stable and repeatable motion. However, higher variability was observed under certain conditions, which may result from partial slipping or the robot being temporarily displaced far from the rotating magnetic field center. In such cases, the MicroTumbler was observed to snap back toward the magnetic axis, introducing abrupt velocity changes and greater measurement uncertainty.

### Precise manipulation of the micro-gripper

The magnetic micro-gripper used in this study builds upon a dual-mode actuation concept developed in our previous work [38]. As illustrated in Fig. 4(a), the gripper consists of two oppositely magnetized blocks embedded at the tips of compliant arms, enabling both controlled deformation and magnetic propulsion. The compliant hinge allows for reversible opening and closing in response to external magnetic torques, while a central through-hole at the end effector expands its ability to grasp and manipulate larger objects. Gradient magnetic fileds are used for locomotion.

To characterize the gripping response, we applied magnetic fields of increasing strength and measured the resulting gripper opening. The experimental setup was consistent with that used for the swimmer experiments (Fig. 2(b)). The gripper was suspended in silicone oil (40 cSt, Alfa Aesar) within a sample holder, and magnetic fields were applied using the MFG-100i system. Actuation and manipulation were recorded using a FLIR camera mounted above the chamber and illuminated by a fluorescent ring light. As shown in Fig. 4(b), the gripper exhibited a progressive increase in opening displacement. This range demonstrates the gripper’s capability to dynamically adapt to objects of varying size. Representative micrographs (inset) show the deformation under 2 mT and 20 mT actuation.

A representative demonstration of the gripper’s precise manipulation capabilities is shown in Fig. 4(c). The gripper was used to grasp and rearrange three micro-objects: two spheres (200 $\mu$m and 250 $\mu$m in diameter) and one cube (250 $\mu$m per side). Through a sequence of controlled locomotion and gripping actions, the objects were arranged into a straight line, illustrating the gripper’s ability to perform deterministic object placement with high spatial accuracy.

### Frequency response of the mini-MicroTumblers (MMTs)

Figure 5(a) shows the schematic design and dimensional parameters of the mini-MicroTumbler (MMT), which operates under the same magnetic actuation principle as the MicroTumbler but is fabricated using a sputter-treated magnet. The MMT incorporates a single embedded permanent magnet oriented orthogonally to its geometric axis. When subjected to a rotating magnetic field, the embedded magnet experiences a magnetic torque that induces periodic edge-over-edge tumbling motion, converting rotational actuation into linear translation along the substrate.

The MMT was fabricated using the DiLL method described in Section II. In contrast to the MicroTumbler, which employed a 250 $\mu$m pre-coated magnet, the MMT utilized an uncoated 100 $\mu$m cubic NdFeB magnet that was surface-smoothed via sputtering prior to printing. This treatment effectively improved surface roughness and optical clarity, preventing bubble formation during the two-photon polymerization (TPP) process and ensuring stable polymer encapsulation around the magnet.

Locomotion testing was conducted the same as in the case of the MicroTumbler (Fig. 3(b)). All experiments were conducted in a dry environment and the tumbler was actuated by a rotating magnetic field. The actuation frequency was varied from 3 Hz to 5 Hz, and real-time motion was captured using an inverted microscope equipped with a FLIR digital camera. As shown in Fig. 5(b), the translational velocity of the MMT increased steadily with frequency, showing the repeatability and stability of the tumbling motion. The inset schematic illustrates the direction of the rotating magnetic field, the induced magnetic moment, and the resulting tumbling trajectory.

These results confirm that the sputtering-treated magnet enables reliable micro-scale fabrication and stable magnetic actuation, demonstrating that the direct-printing approach can accommodate both pre-coated and in-house treated magnets. The MicroTumbler thus serves as a validation platform for extending the MagPrint process toward smaller, high-precision microrobotic systems.

## Conclusion

In this study, we proposed and validated a new direct-printing approach for microrobot fabrication that integrates permanent magnets directly into the microrobot structure using two-photon polymerization. This method significantly enhances fabrication reliability by avoiding post-printing magnet insertion and enabling monolithic design integration. We demonstrated the versatility of this approach by fabricating and testing three representative microrobotic systems: a helical microswimmer, a tumbling microrobot, a magnetically actuated micro-gripper, and a mini-MicroTumbler incorporating a sputter-modified magnet for improved material compatibility.

The microswimmer, printed with an embedded 250 $\mu$m magnet, showed peak forward propulsion at 80 Hz in a viscous medium and revealed clear step-out behavior at higher frequencies, offering insights for optimizing swimming performance in real fluids. The tumbling microrobot exhibited stable locomotion across varied terrain inclinations under low-frequency magnetic actuation, with repeatable and controllable motion suitable for surface-based targeted delivery. The compliant micro-gripper demonstrated dual-mode magnetic actuation, with gripper opening precisely controlled by uniform fields and locomotion enabled by field gradients. Its successful manipulation and alignment of diverse micro-objects validated the platform’s dexterity and reliability.

To extend the fabrication method to uncoated magnets, we introduced a sputtering-based surface modification process that improves the chemical compatibility between magnetic materials and photoresists. This modification enables reliable encapsulation of uncoated magnets without compromising magnetic performance. The successful fabrication of the mini-MicroTumbler demonstrates that the proposed approach is compatible with both pre-coated and surface-modified magnets, greatly enhancing material flexibility and broadening the applicability of the direct-printing process to diverse microrobotic platforms.

There are some limitations on magnet size and fabrication resolution. The minimum magnet size supported by the proposed fabrication approach is primarily constrained by a combination of fixture alignment accuracy, handling capability, and material–process interactions. In principle, the method can accommodate the smallest commercially available micro-magnets, as the printing resolution of TPP is sufficient for sub-100 $\mu$m feature sizes. However, in practice, several limiting factors arise. First, manual handling and placement of magnets become increasingly challenging below 100 $\mu$m due to limited manipulation precision and strong magnetic interactions. Second, fixture fabrication tolerances and alignment accuracy impose constraints on the reliable positioning of smaller magnets within the predefined molds. Third, surface effects, including resin wetting and bubble formation, become more pronounced at smaller scales, potentially affecting print fidelity. From an experimental perspective, further miniaturization also introduces challenges in optical tracking and characterization, as smaller microrobots become difficult to reliably visualize and quantify using standard imaging systems. Therefore, while the proposed method is not fundamentally limited by TPP resolution, the practical lower bound is currently governed by handling, alignment, and measurement constraints, rather than fabrication capability alone.

While in this paper, we primarily demonstrated the capability to fabricate a single microrobot at a time, the proposed direct-printing strategy provides a pathway toward automated batch fabrication through the use of fixture-based magnet arrays. In the current implementation, the fabrication time for a single microrobot (e.g., microswimmer) is approximately 55 minutes, while printing an array of nine devices in a single job may require over 8 hours, including stage movement and alignment time in the Nanoscribe system. Although the total fabrication time remains substantial, the use of pre-aligned fixture arrays can enable fully automated printing without continuous operator intervention, improving reproducibility and reducing manual handling. Therefore, rather than demonstrating high-throughput production at this stage, our results highlight the feasibility of structured, unattended batch fabrication, which can be further optimized in future work through process parallelization and hardware improvements. The proposed fixture-based framework also provides a pathway toward increasingly complex microrobot architectures through programmable magnet placement and multi-material integration.

Overall, our results establish the direct magnet-embedding strategy as a robust and flexible solution for developing complex, multi-functional microrobots. Future work will focus on improving fabrication efficiency, optimizing batch-processing workflows, and further miniaturizing magnetic components through particle-based or thin-film integration to reduce actuation requirements and expand applicability in in vivo environments. Additionally, systematic evaluation of long-term durability will be conducted, including cyclic actuation testing and interfacial stability analysis under repeated loading conditions.

## Data Availability

No datasets were generated or analysed during the current study.
